# Modulating effect of salivary pellicle conditioning of titanium surfaces on the development of biofilms of *Pseudomonas aeruginosa* and *Staphylococcus* species

**DOI:** 10.1038/s41598-026-55672-w

**Published:** 2026-06-03

**Authors:** Miryam Martínez-Hernández, Polet Reyes-Mendoza, Mariana Chávez-Esparza, Adriana-Patricia Rodríguez-Hernández, Víctor I. García-Pérez

**Affiliations:** 1 Biointerfases Laboratory, Division of Graduate Studies and Research, Faculty of Dentistry, National Autonomous University of Mexico (UNAM), Circuito Exterior s/n, Ciudad Universitaria, 04510 Mexico City, Mexico; 2 Molecular Genetics Laboratory, Division of Graduate Studies and Research, Faculty of Dentistry, National Autonomous University of Mexico (UNAM), Circuito Exterior s/n, Ciudad Universitaria, 04510 Mexico City, Mexico

**Keywords:** Salivary pellicle, Titanium surfaces, Dental implants, *Staphylococcus aureus*, *Staphylococcus epidermidis*, *Pseudomonas aeruginosa*, Biofilms, Cellular microbiology, Oral diseases

## Abstract

To evaluate the modulating effect of salivary pellicle (SP) on titanium (Ti) surfaces on the development of *Staphylococcus* species and *Pseudomonas aeruginosa* biofilm. Ti substrates were incubated for 2 h with whole saliva samples to induce the salivary pellicle formation. After conditioning with SP, Ti substrates were incubated for 12 h with *P. aeruginosa*, *S. aureus*, *S. epidermidis* strains individually, in addition to co-culture of the three bacteria. The biofilm development on the Ti substrates was visualized by Scanning Electron Microscopy (SEM). To measure the metabolic activity and vitality of cells within the biofilm the XTT assay was used, while the proportion of the species tested in the biofilms was determined throughout DNA-DNA hybridizations. SP modulated the biofilm development on the Ti surfaces, favoring the formation of *P. aeruginosa* biofilms, while inhibiting the growth of *S. aureus* and *S. epidermidis*. In the case of the coculture biofilms, a predominance of *P. aeruginosa* cells over the *Staphylococcus* strains was observed. When the bacterial strains were tested individually, the SP importantly reduced the development of biofilms of *Staphylococcus* species, whereas it favored the development of *P. aeruginosa* biofilms on Ti surfaces. Similarly, when mixed biofilms developed, SP favored the selective expansion of *P. aeruginosa* and *S. aureus* growth over *S. epidermidis* growth. The results of this study provide valuable information on the modulatory effect of the SP on the development of opportunistic bacteria biofilms on Ti surfaces used for dental and oral implantology.

**Supplementary Information:**

The online version contains supplementary material available at 10.1038/s41598-026-55672-w.

## Introduction

Biofilm formation on biomedical surfaces is always a concern during surgeries involving the oral cavity, primarily because the oral cavity harbors one of the largest and most diverse microbiotas of the body, comprising over 800 different bacterial species^1,2^. The vast majority of the oral cavity bacteria exist in the form of biofilms.

In general, dental biofilm maintains a harmonious relationship with the host, providing benefits to overall health^3,4^, nevertheless, alterations in biofilm composition caused by certain stress factors, such as tobacco use or lack of plaque removal, can alter the biofilm homeostasis, leading to the development of oral diseases, such as periodontitis or peri-implantitis^5^.

Peri-implantitis is the main biological complication that can negatively affect long-term outcomes of the osseointegrated dental implants^6^. Even though the long-term survival rates of dental implants are high (≥ 10 years)^7^, it is estimated that approximately 1/3 of all patients and 1/5 of all implants placed will experience peri-implantitis^8^.

The peri-implant infections have often been regarded as pathologies analogous to gingivitis and periodontitis, mainly because those pathologies share similarities in clinical characteristics and aetiology^9^. However, there is increasing evidence supporting the notion that periimplantitis is not periodontitis^10^, such as critical histopathological differences between peri-implantitis and periodontitis have been found^11^, in addition to current microbial identification methods have revealed an unexpected difference between the microbiome surrounding teeth and that surrounding implants^12^.

Regarding the characterization of the microbiota associated with the presence of peri-implant damage, through the use of DNA probes and culture analysis, it has been possible to isolate common periodontopathogenic bacteria in diseased peri-implant sites, including *Porphyromonas gingivalis* and *Tannerella forsythia*^13^. However, it has recently been reported that the development of peri-implant lesions could be associated with bacterial species other than canonical bacteria associated with periodontitis, namely *P. gingivalis*, *Treponema denticola*, and *T. forsythia*^14^. In this regard, it has been pointed out that the presence, in high proportions, of pathogenic-opportunistic and antibiotic-resistant bacteria such as *P. aeruginosa*, *S. aureus*, and *S. epidermidis* could play an important role in the etiopathogenesis of peri-implantitis^15–18^. These reports indicate that the presence of such bacterial strains on dental implant surfaces is due to a certain “affinity for Titanium (Ti)”^15,19,20^. However, the underlying mechanisms of this affinity have not been explored in depth.

Ti surfaces of dental implants *vs.* cementum or natural tooth enamel represent chemically different substrates; differences in surface energy, topography, wettability, and electrochemical charges of these substrates will modulate bacterial adhesion, ultimately resulting in the formation of distinct biofilms on the different substrates^21^. An essential prerequisite for bacterial adhesion and subsequent biofilm development in the oral cavity is salivary pellicle (SP) formation. SP is a thin film coating all oral surfaces, and mainly consists of amino and fatty acids, proteins, and carbohydrates derived mainly from saliva, but also from microorganisms^22,23^. The formation of SP involves a specific and non-random complex process that will result in the selective adsorption of biomolecules^24^. The SP formed on Ti surfaces has aroused great interest due to its importance in peri-implant health^25,26^.

SP formed on dental enamel, and the one formed on titanium surfaces has shown important molecular differences^27^, which could explain the presence of *S. epidermidis*, *S. aureus*^15,17,28^, and *P. aeruginosa*^29–31^ in peri-implantitis sites. In a study by El-Telbany, *et al.*^31^. it was possible to isolate *P. aeruginosa* in 10% of cases of peri-implantitis evaluated, indicating that the presence of these pathogens would be associated with a higher prevalence of disease^32^.

Since the modulating effect of SP formed on titanium surfaces in the formation of oral biofilms is now recognized^33^, a deeper understanding of how this protein film affects the adhesion of non-canonical periodontal pathogens, such as the opportunistic pathogens *Staphylococcus* species and *P. aeruginosa*, to which the aetiology of peri-implantitis has even been attributed, is now needed.

## Materials and methods

### Ti surfaces tested

Ti disks with a diameter of 15 mm were prepared from 1-mm-thick sheets of grade 2 unalloyed Ti (ASTM F67). The methods used to produce the pretreatment (PT) surfaces have been previously reported^27^. The Ti surfaces are relatively smooth with an average roughness of (Ra) < 0.19 μm and a water contact angle of 81.9° ± 0.4°. Ti disks were manufactured by Institut Straumann AG (Basel, Switzerland) and delivered to us ready to use. For each experiment, the number of Ti disks was adjusted to normalize the total surface area evaluated to 8 cm^2^.

### Salivary pellicle formation assays

Unstimulated whole human saliva samples used for salivary pellicle formation assays on titanium substrates were obtained from a healthy, middle-aged volunteer with no active caries lesions or history of periodontal disease. The healthy saliva donor was currently a nonsmoker, with more than 20 natural teeth . Clinical measurements were taken from the healthy volunteer at six sites per tooth (mesiobuccal, buccal, distobuccal, distolingual, lingual, and mesiolingual) at all teeth excluding third molars (approximately 168 sites evaluated) as previously described^34^.

Saliva samples were collected between 8:00 am and 10:00 am. The volunteer was asked to refrain from eating, drinking, or performing oral hygiene procedures at least 12 h before saliva collection. Before saliva samples were collected, the volunteer was asked to rinse their mouth with clean water for 30 s to remove desquamated epithelial cells, microorganisms, and food debris. Sterile 50 ml tubes were used to collect passive drooling saliva for 3 min. Saliva samples were centrifuged at 15,000 g, 4 °C, for 15 min; the resulting clean supernatants (without being filtered) supplemented with cOmplete™, EDTA-free Protease Inhibitor Cocktail (RocheDiagnostics, Penzberg, Germany; Cat. No. 04 693 132 001), were used for salivary pellicle formation assays on Ti substrates. The saliva donor provided written informed consent acknowledging his willingness to participate. This study was performed under the guidelines of the Declaration of Helsinki and approved by the Ethics Committee for Human Studies of the Division of Postgraduate Studies and Research, School of Dentistry, National Autonomous University of Mexico (CIE/0108/09/2023).

To induce salivary pellicle formation, Ti surfaces were placed individually in 24-well plates with 500 µL of whole saliva and incubated for two hours at 37 °C in a chamber with 10% humidity. After incubation, the surfaces were washed twice with 1 mL of bidistilled water (H_2_O_dd_) to remove non-adsorbed proteins. Another set of surfaces incubated under the same conditions without salivary film formation was used as a control. Subsequently, the surfaces were transferred to new sterile 24-well cell culture plates and were immediately used in biofilm development assays.

### Biofilm development assays of *Staphylococcus* species and *P. aeruginosa*

Three ATCC reference strains were used for *in vitro *biofilm development assays on the tested surfaces (Table 1). All strains were obtained as lyophilized cultures from the American Type Culture Collection (ATCC, Rockville, MD, USA).

**Table 1:** Reference strains used for the biofilm development assays.

Specie	Strain^a^	Gram
*S. aureus*	25923	Positive
*S. epidermidis*	14990	
*P. aeruginosa*	43636	Negative

^a^American Type Culture Collection, Rockville, MD, USA.

Each strain was grown on enriched Trypticase Soy Agar (TSA; BBL, Becton Dickinson) supplemented with 0.3 µg/mL menadione (Sigma-Aldrich) and 5 µg/mL hemin (Sigma-Aldrich) for 24 h at 37 °C under aerobic conditions. After the incubation period, the different bacterial cultures were harvested and individually suspended in tubes containing enriched TSB broth (TSB added with 0.3 µg/mL menadione and 5 µg/mL hemin). The optical density (OD) in each tube was adjusted to 1.0 at λ = 600 nm using a spectrophotometer (FilterMax F5 Multi-Mode Microplate Reader, Thermo Fisher) to obtain a bacterial stock suspension containing 1 × 10^9^ cells/mL. To evaluate biofilm development, Ti surfaces—with or without salivary pellicle—were incubated with bacterial suspensions adjusted to 1 × 10⁶ cells/mL, either individually or in co‑culture. Cultures were incubated for 12 h at 37 °C on an orbital shaker (~ 160 rpm). After incubation, surfaces were washed twice with enriched broth to remove nonadherent bacteria. All experiments were performed in triplicate and repeated at least twice independently.

### Scanning electron microscopy (SEM) observations

The microscopic appearance of the biofilm of *S. aureus*, *S. epidermidis*, and *P. aeruginosa* or bacterial coculture developed on Ti surfaces covered and uncovered (control surfaces) by the salivary pellicle, was observed by SEM. In brief, samples were fixed in 2% glutaraldehyde (commercial solution, Gafidex^®^ without activator) for 24 h at room temperature, washed three times in phosphate-buffered solution (PBS, pH 7.4), and dehydrated through graded ethanol (20–100%). Specimens were stored in a desiccator for 24 h, sputter-coated with gold (SDC 050; Bal-Tec AG, Balzers, Germany), mounted on SEM stubs, and imaged using a Scanning Electron Microscope (SEM, JEOL JSM5600-LV, Japan) operated at 5 kV in high‑vacuum mode. Biofilm coverage on each surface was quantified using pixel‑based analysis in ImageJ.

### Viability analysis

The viability of the biofilm cells of *S. aureus*, *S. epidermidis*, and *P. aeruginosa*, as well as the co-culture of these strains, on salivary pellicle-covered or uncovered Ti surfaces was assessed by using the XTT (2,3-bis(2-methoxy-4-nitro-5-sulfophenyl)-2 H-tetrazolium-5-carboxanilide) assay (Sigma, MO, USA). Briefly, after aerobic incubation, the inoculated surfaces were rinsed twice with PBS to remove non-adhered bacteria and transferred to sterile well plates. Then, the surfaces were incubated for 3 h in 1 mL of enriched TSB broth added with 20 µL of the XTT solution. Finally, the O.D. of 100 µL aliquots of the supernatants was read at λ = 450 nm in a FilterMaxF5 multi-mode microplate reader (Molecular Devices, USA), and the number of viable bacteria was calculated according to standard curves previously performed. Standard curves were made for each species separately and for the co-culture. Three replicates were performed on three separate occasions to obtain the viability analysis results.

### Checkerboard analysis

After aerobic bacterial incubation, each Ti specimen was washed twice with TSB broth. Subsequently, 1 ml of enriched broth was added, and samples were sonicated for 5 periods of 10 s each to detach the bacteria adherent.

To determine the proportion of each bacterial strain of the biofilm developed on the Ti surfaces, 100 µl of the bacterial suspension obtained after sonication of each sample was transferred to individual Eppendorf tubes with 100 µl of 0.5 *M* NaOH. The bacterial species comprising the developed biofilm were identified and quantified using the Checkerboard DNA–DNA hybridization technique previously described^34,35^. Briefly, whole-genomic DNA probes were prepared using the growth from 3- to 7-day cultures of the three reference strains used for the biofilm development assays (Table 1). Bacterial cells were harvested and placed in tubes containing 1 mL of TE buffer, washed twice, and lysed at 37 °C for 1 h. Gram-negative cells were lysed using 10% Sodium Dodecyl Sulfate (SDS, Sigma-Aldrich) plus proteinase K (20 mg/ml; Sigma-Aldrich), whereas Gram-positive cells were lysed using lysozyme (15 mg/mL; Sigma-Aldrich) plus achromopeptidase (5 mg/ml; Sigma-Aldrich). DNA was isolated and purified. Whole-genomic DNA probes were prepared for each species by labeling 1 mg of DNA with digoxigenin (Roche Diagnostics, Mannheim, Germany) using the random primer technique^36^. The sensitivity of the assay was set to allow the detection of approximately 10^4^ to 10^7^ cells of a given species by adjusting the concentration of each DNA probe. For the DNA–DNA hybridization, each sample was thawed at room temperature, boiled for 10 min, and neutralized with 800 µl 5 *M* ammonium acetate (Sigma-Aldrich). The released DNA from each sample was then placed into individual lanes (Min- islot-30, Immunetics, Cambridge, MA), concentrated onto a 15 × 15 cm positively charged nylon membrane (Roche Diagnostics), and fixed to the membrane by cross-linking under ultraviolet light. Two lanes on each membrane contained standards consisting of a mixture of 10^5^ and 10^6^ cells of each bacterial species tested. The membranes were prehybridized at 42 °C for 2 h and placed in a second device (Miniblotter-45, Immunetics) with the sample lanes rotated 90º to the channels of the apparatus. Probes were hybridized in two sets of three consecutive channels, leaving empty channels (hybridization solution only) to allow noise and background correction of signals. The membranes were washed twice at high stringency for 20 min each time at 68 °C in phosphate buffer (0.1X SSC and 0.1% SDS). Membranes were blocked by 1-h incubation in a blocking buffer containing 1% casein in maleate buffer [100 m*M* maleic acid (Sigma-Aldrich), 150 m*M* NaCl, pH 7.5]. Hybrids were detected by exposing the membranes to a 1:50,000 dilution of antidigoxigenin antibody conjugated to alkaline phosphatase (Roche Diagnostics) for 30 min. Signals were detected by chemiluminescence using a chemiluminescent agent (CDP-Star, Roche Diagnostics) for 30 min on the membranes at room temperature and exposed to film in autoradiographic cassettes for 30 min. Signals were detected with specialized software (Quantity One, BioRad Laboratories), adjusted by subtracting the average plus two standard deviations of the noise and background detected in the empty lanes, and converted to absolute counts by comparison with the standards on the membrane. Failure to detect a signal was recorded as zero.

### Statistical analysis

Data for XTT values and total bacterial counts are presented as the Mean ± Standard Error of the Mean (SEM). All data were analyzed by Student´s t-test using Bonferroni´s correction using IBM-SPSS 21 software. Statistical significance was set at *p* *≤* 0.05.

For data derived from the Checkerboard technique, bacterial counts were determined using reference standards of 10⁵ and 10⁶ cells and expressed as individual mean levels × 10⁵ cells. The proportional data for each species (*S. aureus*, *S. epidermidis*, *P. aeruginosa*, and their co-culture) of the total DNA probe count were expressed as the mean proportion of each species (% of total DNA probe count).

## Results

In the present work, the modulating effect of the salivary film formed on titanium surfaces on the development of biofilms of *Staphylococcus* species and *P. aeruginosa* was investigated.

### Saliva donor

For the present investigation, a donor subject was selected to reduce the variability in the protein profile that could result from the inclusion of different volunteers, as has been previously reported^37^. The volunteer underwent a full periodontal evaluation to confirm the diagnosis of periodontal health, which he defined as the absence of sites with probing depths ≤3 mm and bleeding values at full-mouth probing < 10%^5^. The results of the full periodontal evaluation are presented in a Supplementary Table 1.

### Biofilm morphology evaluated by SEM

**Fig. 1:**
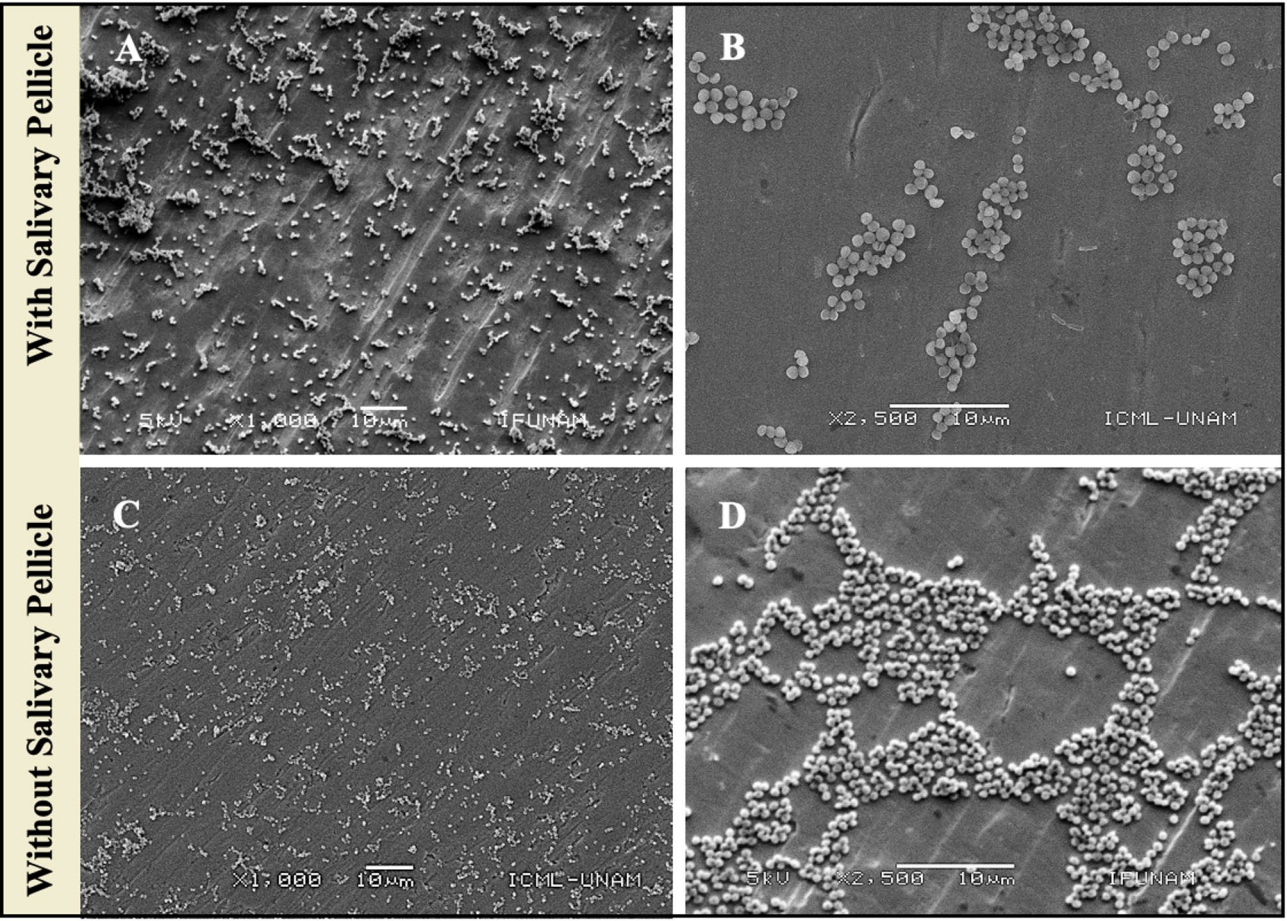
*S. aureus* biofilm developed on Ti surfaces. (**A**). Biofilm developed on Ti surfaces conditioned with salivary pellicle (magnification x1000), and (**B**). a higher magnification (x2500). (**C**). Biofilm developed on unconditioned Ti surfaces (magnification x1000), and (**D**). a higher magnification (x2500).

**Fig. 2:**
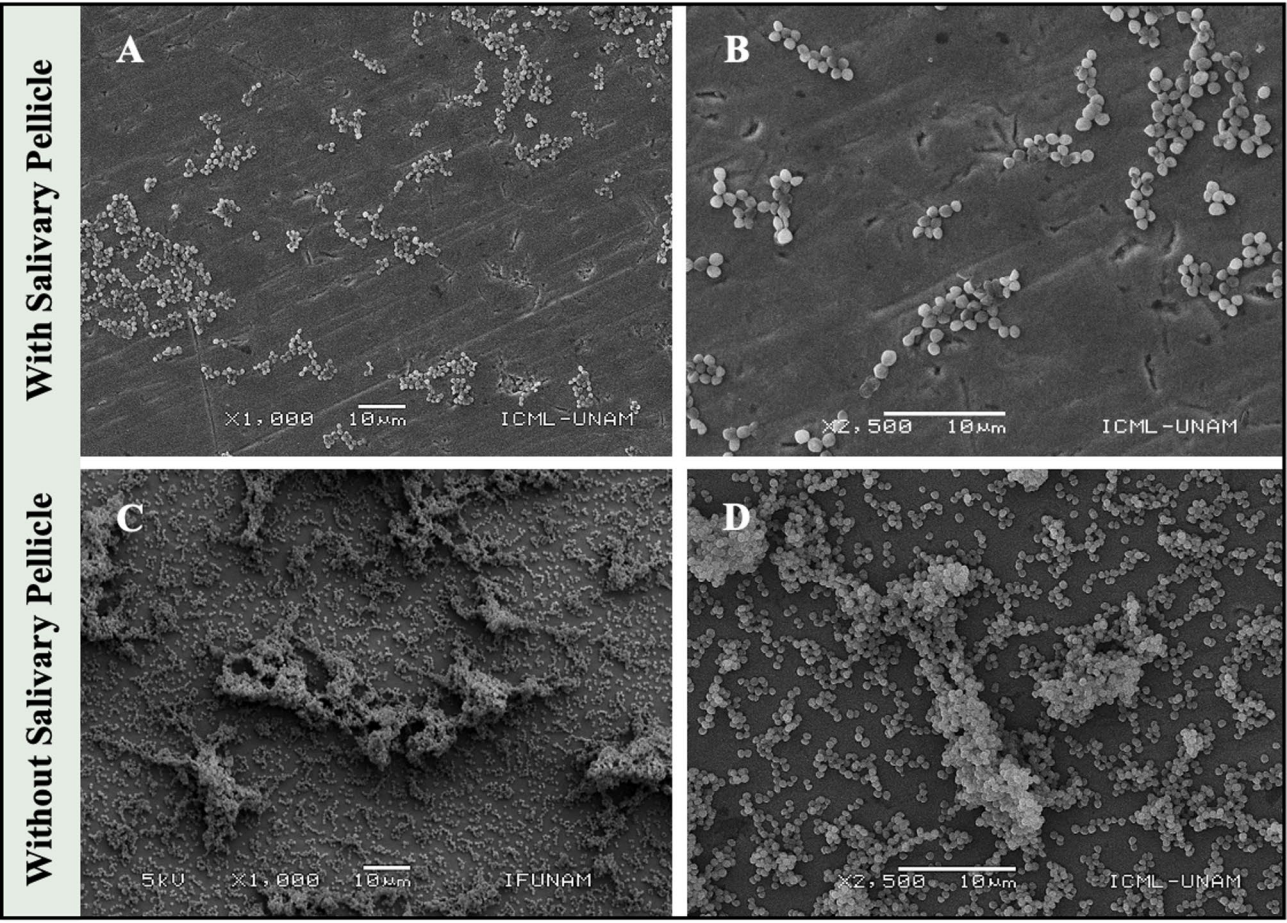
*S. epidermidis* biofilm developed on Ti surfaces. (**A**) Biofilm developed on Ti surfaces conditioned with salivary pellicle (magnification x1000), and (**B**) a higher magnification (x2500). (**C**) Biofilm developed on unconditioned Ti surfaces (magnification x1000), a (**D**) a higher magnification (x2500).

**Fig. 3:**
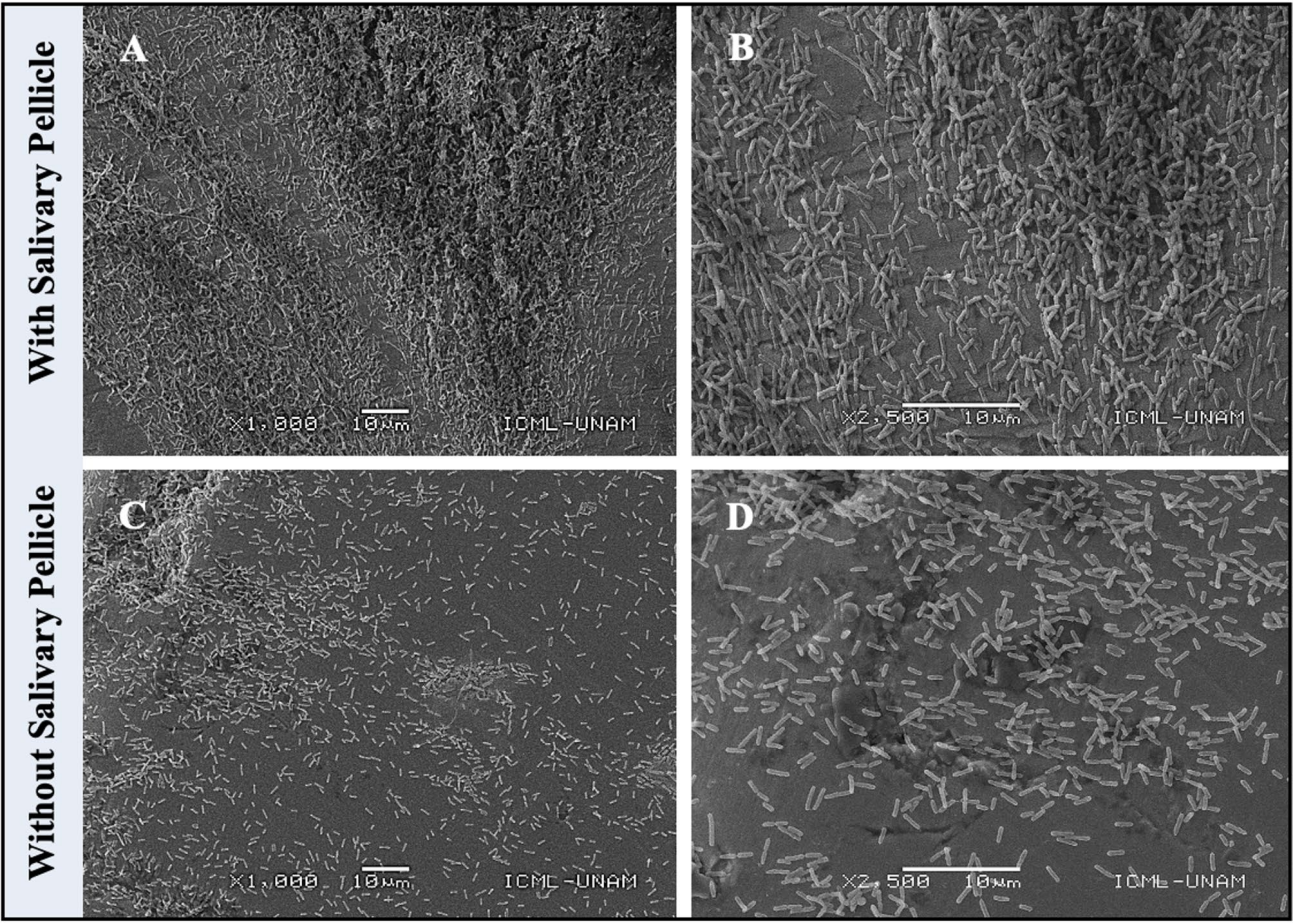
*P. aeruginosa* biofilm developed on Ti surfaces. (**A**) Biofilm developed on Ti surfaces conditioned with salivary pellicle (magnification x1000), and (**B**) a higher magnification (x2500). (**C**) Biofilm developed on unconditioned Ti surfaces (magnification x1000); and (**D**) a higher magnification (x2500).

**Fig. 4:**
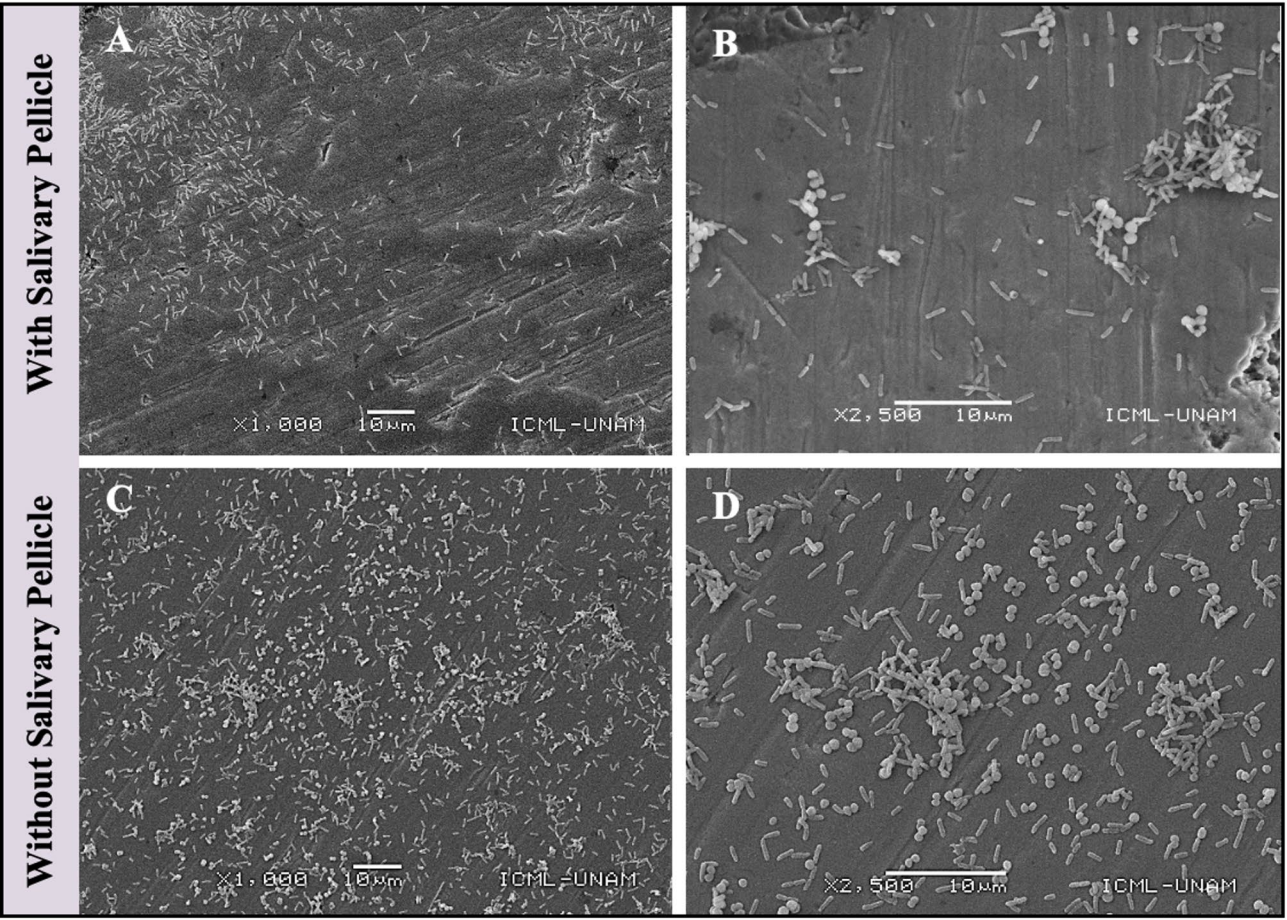
*S. aureus*, *S. epidermidis*, and *P. aeruginosa* coculture biofilm developed on Ti surfaces. (**A**) Biofilm developed on Ti surfaces conditioned with salivary pellicle (magnification x1000), and (**B**) a higher magnification (x2500). (**C**) Biofilm developed on unconditioned Ti surfaces (magnification x1000), and (**D**) a higher magnification (x2500).

The following images were obtained by SEM using secondary electrons of biofilms developed by *S. aureus*, *S. epidermidis*, *P. aeruginosa*, as well as their coculture developed on Ti surfaces conditioned with SP (experimental) and unconditioned by SP (control) (Figs. 1, 2, 3 and 4).

SEM analysis showed comparable *S. aureus* biofilm morphology on both salivary pellicle-conditioned and unconditioned Ti surfaces. In SEM micrograph 1A, corresponding to the Ti surfaces conditioned with the salivary film, it is possible to observe *S. aureus* cells forming grape-like clusters, periodically distributed on the conditioned substrate. However, on these surfaces, it was not possible to observe the formation of a highly complex biofilm; this finding can be seen in greater detail mainly in image 1B. Regarding the control Ti substrates (not conditioned with the SP), images 1C and 1D demonstrate that bacteria adhere to these surfaces in a regular pattern, with clusters of cocci covering the surface.

The development of *S. epidermidis* biofilms was higher on the control substrates (Fig. 2), i.e., those that were not conditioned with the SP. On the Ti surfaces that were conditioned with the SP (images 2A and 2B), a few bacterial clusters can be seen adhering to the conditioned surface. In contrast, unconditioned Ti surfaces exhibited markedly greater bacterial adhesion, abundant exopolysaccharide production, and more structurally complex biofilms covering a larger surface area (images 2C and 2D).

In Fig. 3, it is possible to observe the biofilm developed by *P. aeruginosa* on the Ti surfaces conditioned with the salivary pellicle compared to the biofilm of *P. aeruginosa* developed on Ti surfaces that were not conditioned with the PS (control). As can be seen in images 3A and 3B, a higher number of bacterial cells adhered to the surfaces, along with the formation of a complex biofilm, compared to the unconditioned surfaces (images 3C and 3D), where biofilm development was minor.

Figure 4 shows the representative images of biofilm formation developed by the co-culture of *P. aeruginosa*, *S. aureus*, and *S. epidermidis*, in which it is possible to observe the differences in biofilm formation on the Ti surfaces conditioned with the salivary pellicle compared to the biofilms formed on the Ti surfaces not conditioned with the SP. As can be seen, in terms of the number of cells adhering to the surfaces and in the percentage of area covered by biofilm, it is not possible to identify differences in bacterial adhesion on the substrates conditioned with the SP (images 4A and 4B) compared to the substrates that were not conditioned with the saliva pellicle (control). However, regarding the bacterial species predominantly colonizing the surfaces, there was an important difference, on the surfaces conditioned with the salivary film (image 4A, B) a predominance in the adhesion of *P. aeruginosa* (bacilli), with few cocci adhering to the surface, while on the surfaces that were not conditioned by the SP, it was possible to observe the presence of a greater number of bacterial cells in the form of cocci, which would correspond to a greater adhesion of *S. aureus* and/or *S. epidermidis* to these samples. For additional representative images, see the Supplementary Fig. 1.

To corroborate the qualitative morphological observations described above, a quantitative pixel analysis was performed to estimate the percentage of titanium surface area covered by the biofilm (see Supplementary Table 2). The analysis confirmed distinct colonization patterns modulated by the salivary pellicle. While the presence of the pellicle resulted in comparable surface coverage for *S. aureus* (≈ 20%) and the mixed-species co-cultures (≈ 42 − 50%) relative to controls, it exerted a marked influence on the other species. Specifically, the salivary pellicle drastically reduced the surface coverage of *S. epidermidis* (≈ 18% vs. ≈91% on unconditioned surfaces), whereas it appeared to favor the colonization of *P. aeruginosa*, which exhibited near-total confluence (98%) on the conditioned substrates compared to the control (71%).

### Biofilm metabolic activity evaluated by XTT assay

**Fig. 5:**
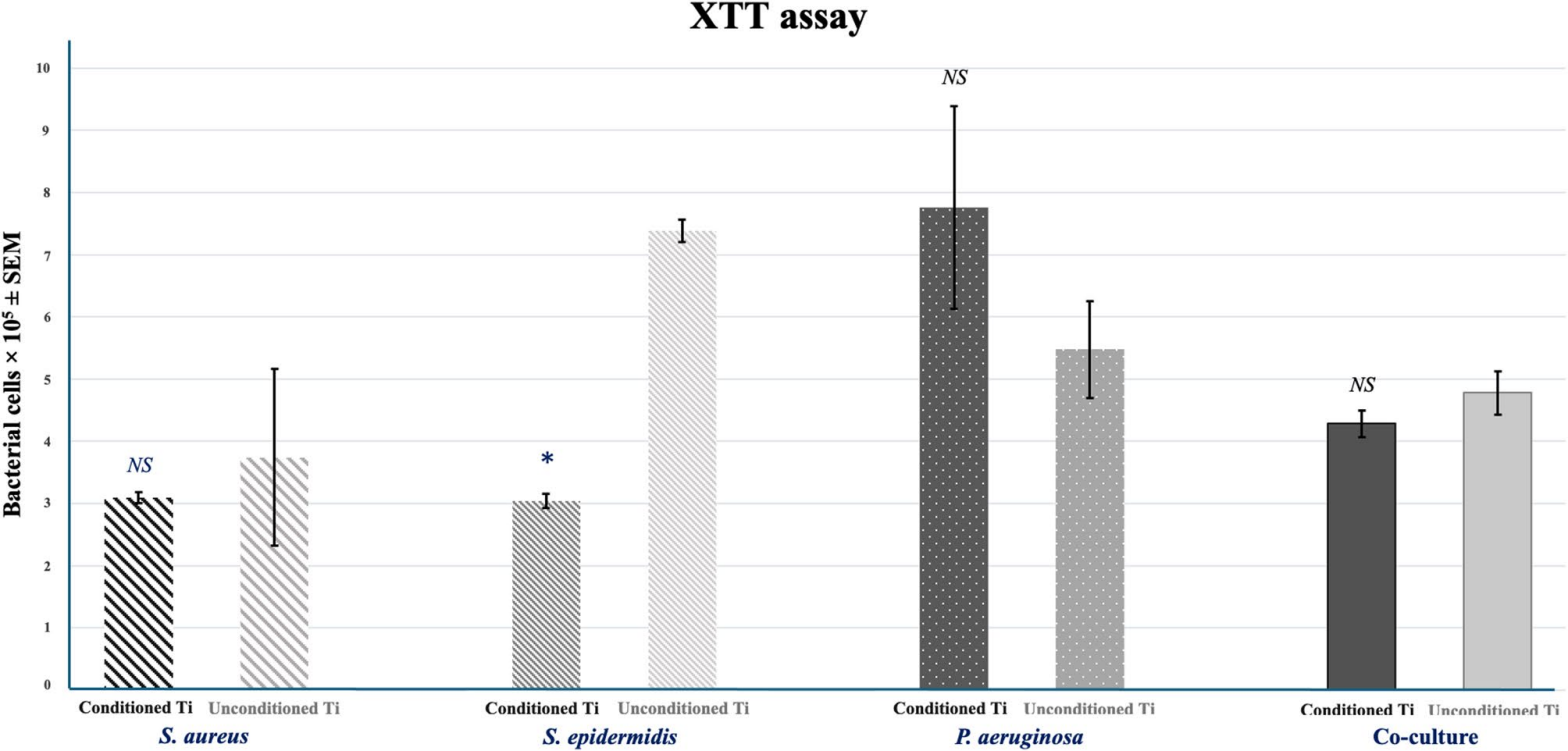
Viable bacterial counts on titanium surfaces assessed by XTT metabolic assay. The bar chart represents the mean number of viable bacterial cells (× 10^5^ ± SEM, standard error of the mean) for *S. aureus*, *S. epidermidis*, *P. aeruginosa*, and their co-culture. Viability was determined by converting OD values from the XTT assay using standard curves generated for each species. Biofilms were developed on titanium surfaces conditioned with salivary pellicle (Conditioned Ti) or unconditioned controls (Unconditioned Ti). Comparisons between conditioned and unconditioned surfaces: *NS*, no significant; ^*^p < 0.01.

Optical density (OD) values representing bacterial viability in the XTT assay are shown in Fig. 5. The figure shows the results of the biofilm developed by each of the bacteria separately, in addition to the three bacterial species evaluated in coculture on the Ti surfaces conditioned with the salivary pellicle.

A similar trend was observed in the metabolic activity of the biofilms composed of *Staphylococcus* species on Ti surfaces. That is, the presence of the salivary pellicle on the substrates reduced the bacterial viability of both strains. The presence of SP reduced the viability of *S. aureus* cells attached to Ti surfaces; this was compared to that observed on substrates that were not conditioned. In general, *S. epidermidis* biofilm development was found to be higher than that of *S. aureus* on the surfaces tested. Furthermore, the presence of a salivary pellicle on Ti substrates had been demonstrated to reduce the viability of *S. epidermidis* bacteria, as opposed to that observed for unconditioned substrates, respectively (*p* < 0.01).

When evaluated in isolation, *P. aeruginosa* demonstrated the highest overall affinity for the substrate, irrespective of prior conditioning. While the presence of the salivary pellicle yielded a higher mean viability for *P. aeruginosa* relative to the unconditioned control, the data did not reach statistical significance (*NS*).

Finally, when biofilm formation of *P. aeruginosa*, *S. aureus*, and *S. epidermidis* was evaluated in co-culture, it was found that there was less bacterial adhesion on surfaces that were coated with the salivary pellicle compared to the adhesion observed on surfaces that were not coated with the PS; however, this difference was not significant. Furthermore, it is not possible to attribute this reduction solely to the effect of metabolic interactions. The results derived from counting the Colony-Forming Units (CFUs) formed by bacteria detached from the surface of the Ti samples analyzed are shown in Supplementary Fig. 2.

### Biofilm composition evaluated by DNA-DNA hybridizations

**Fig. 6:**
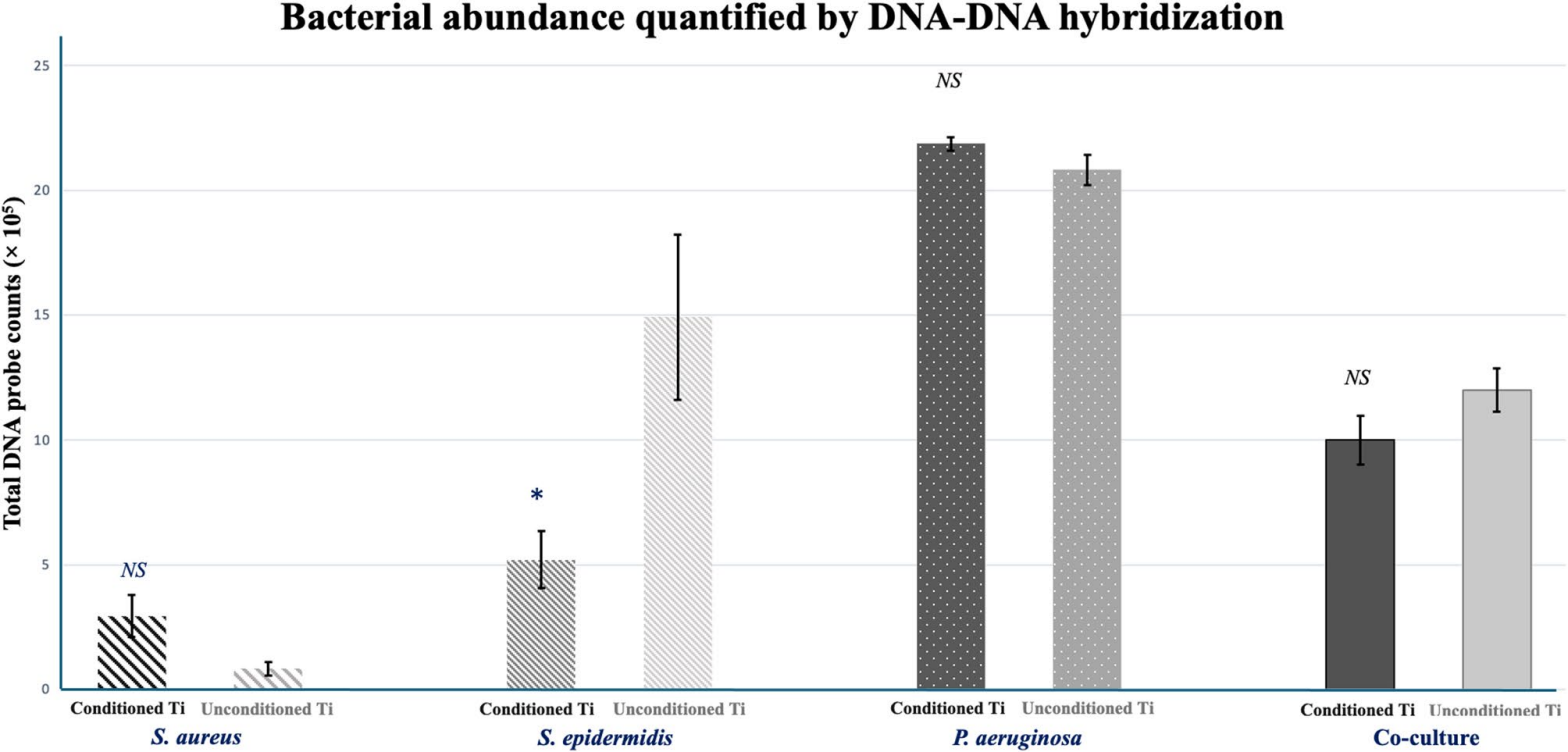
Bacterial abundance quantified by DNA-DNA hybridization. This graph displays the mean individual levels (total DNA probe count × 10^5^ ± SEM, Standard Error of the Mean) for single-species biofilms and the total bacterial load within the mixed-species co-culture. Biofilms were developed on titanium surfaces conditioned with salivary pellicle (Conditioned Ti) or unconditioned controls (Unconditioned Ti). Comparisons between conditioned and unconditioned surfaces: *NS*, no significant; ^*^p < 0.01.

To determine the proportion of each bacterial strain that adhered to the Ti surfaces, DNA-DNA hybridizations were performed. Different patterns of biofilm formation were observed on the Ti disk conditioned or unconditioned by the SP pellicle (Fig. 6).

Biofilm development on the titanium substrates exhibited distinct trends contingent upon both the bacterial species and the presence of salivary pellicle conditioning (Fig. 6). For *S. aureus*, the mean number of colonizing cells demonstrated an important numerical increase on the conditioned surfaces compared to the unconditioned controls, rising from 0.8 × 10^5^ ± 0.3 to 3.0 × 10^5^ ± 0.9. However, this difference did not reach statistical significance (*NS*). For *S. epidermis*, total DNA probe counts correlated with the level of cellular metabolic activity (as indicated by the XTT assay result). Lower counts were observed on Ti surfaces conditioned with the salivary film than on unconditioned Ti discs, confirming that the protein film exerted an inhibitory effect on this bacterial strain (5.2 × 10^5^ ± 1.1 *vs. *14.9 × 10^5^ ± 3.3) (*p* < 0.01).

In contrast to the dynamics observed for the *S. epidermidis* biofilm, total DNA probe counts for *P. aeruginosa* were numerically higher on substrates conditioned with the salivary pellicle compared to the unconditioned titanium controls. Although both DNA quantification and metabolic assays suggested an increase in *P. aeruginosa* colonization on the conditioned surfaces, these trends did not reach statistical significance.

Regarding co-culture biofilm, it was found that, although there was minor bacterial adhesion on conditioned surfaces compared to the adhesion observed on surfaces that were not coated with the PS, this difference was not significant.

**Fig. 7:**
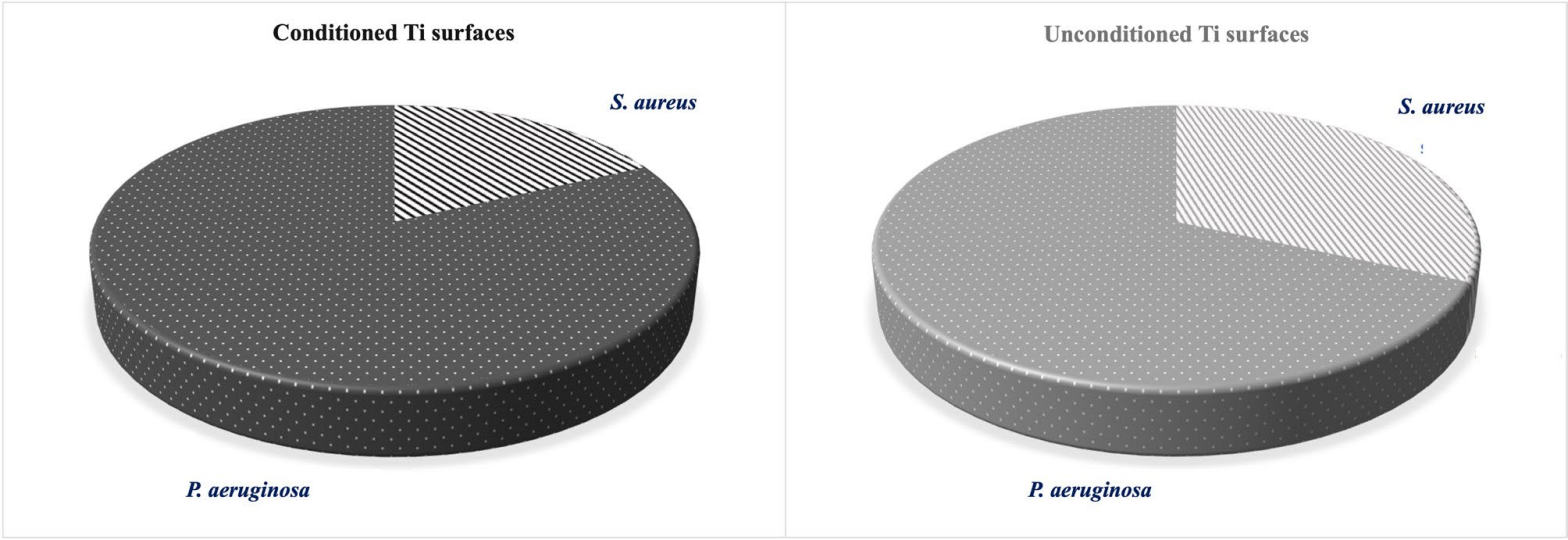
Relative community composition of the co-culture biofilm. Pie charts illustrate the mean proportional distribution (% of total DNA probe count) of bacterial species within the co-culture biofilms formed on salivary pellicle-conditioned *vs.* unconditioned titanium surfaces.

In addition, the mean proportions (% of the total DNA probe count) of the bacterial strains evaluated in coculture on the biofilm developed on the Ti conditioned or unconditioned by the salivary pellicle were analyzed (Fig. 7). As can be seen, no growth of the *S. epidermidis* was detected, which would not necessarily indicate that its growth was entirely suppressed when cocultured with *S. aureus* and *P. aeruginosa*, but rather that its total number was likely below the detection level of the DNA-DNA hybridization technique of 1 × 10^4^. Although the distribution of the biofilms showed a numerical dominance of *P. aeruginosa* over *S. aureus* on both conditioned and bare titanium surfaces (80.4% vs. 19.6% and 68.8% *vs.* 31.2%, respectively), the lack of statistical significance prevents us from confirming a true ecological shift. Instead, this variance simply reflects the information uncertainty inherent to the community dynamics.

## Discussion

Titanium remains the metal of choice in dental implantology due to its physicochemical and biocompatibility properties. Once dental implants are placed in the oral cavity and exposed to biofluids such as blood and saliva, their surface is subject to the adsorption of biomolecules, with the consequent formation of salivary pellicle (SP) on them, modifying their biological behaviors^38^.

In general terms, dental implants exhibit high success rates. However, bacterial interactions and the subsequent biofilm development on the surfaces of these biomedical devices play a key role in the emergence of peri-implant infections, compromising the longevity of the functional dental implants in the oral cavity. There is no clear understanding of the exact mechanism of microbial interaction during peri-implantitis^10,39^. While some studies conclude that the microbial profile is similar to periodontitis^40,41^, contradictory studies have reported that opportunistic bacteria, such as *S. aureus*, which are not part of the most common species in the oral cavity^42^, play a role in the progression of the disease^15,17^. In recent decades, it has been identified that the development of peri-implantitis lesions is associated with the presence, in high proportions, of pathogen-opportunistic and antibiotic-resistant bacteria such as *P. aeruginosa*, *S. aureus*, and *S. epidermidis*^16,43^. Although different reports indicate that they show some affinity for Ti, the underlying mechanisms of this so-called “affinity” have not been explored in depth^19,20^.

Understanding the ecological triggers underlying the microbial pathogenesis of peri-implantitis is essential for the development of better preventive, diagnostic, and therapeutic strategies, since peri-implant tissues are more susceptible to endogenous oral infections. Due to the above, the main objective of the present investigation was to evaluate the modulating effect of the salivary pellicle formed on Ti surfaces on the development of *P. aeruginosa*, *S. aureus*, and *S. epidermidis* biofilms.

According to the overall results obtained through the tests performed in this research: SEM, XTT assay, and DNA-DNA hybridizations, when the biofilm development of bacterial strains was evaluated individually, it was observed that the highest affinity for the substrates, regardless of whether they were conditioned or unconditioned, was for the *P. aeruginos*a strain, followed by *S. epidermidis*, while *S. aureus* showed the lowest biofilm development. The enhanced adhesion and subsequent biofilm development by *P. aeruginosa* on the surfaces tested could be attributed to an increase in the level of extracellular polymeric substance (EPS) produced by this microorganism, as previously reported^19^. *P. aeruginosa* produces distinct types of exopolysaccharides, such as alginate, Pel, and Psl, which may play an important role in biofilm formation^44,45^, while providing a local environment that favors adhesion, bacterial community cohesion, and communication through highly specific interactions between cells of both bacterial and host origin^46^. In clinical settings, *P. aeruginosa* has been isolated from the palate and dorsum of the tongue in a slightly elevated proportion in implant-bearing patients^47^. Given the well‑documented pathogenicity of *P. aeruginosa*, the observation that salivary pellicle enhances rather than inhibits its biofilm formation on Ti surfaces underscores a clinically significant risk factor for implant‑associated infections.

Regarding *Staphylococcus* species biofilm development on the Ti substrates, a marked difference was found between the higher affinity of *S. epidermidis* for the substrates compared to the affinity shown by the *S. aureus* strain. However, this affinity changed as the presence of *S. epidermidis* was masked by *S. aureus* and especially *P. aeruginosa* in the biofilms developed by the strains studied in coculture.

It has previously been reported that *S. epidermidis*, along with *S. aureus*, is isolated in high proportions from Ti dental implant surfaces^48,49^. Indeed, both *S. aureus* and *S. epidermidis* have been associated with subgingival flora around failed implants^16,50^. The adhesion of *S. aureus* to Ti dental implant surfaces is regarded as favored by the chemical characteristics of the substrate^51^. In fact, *S. aureus* is frequently isolated from sites with peri-implantitis^52^, and is associated with implant loss^32^.

The finding that the conditioning of the Ti substrates with the PS only had a modulating effect by reducing the growth of *S. epidermidis*, whereas it had no significant effect on the development of *S. aureus* or *P. aeruginosa* biofilms when evaluated individually contrast with earlier reports^33^, which showed that the salivary pellicle formed on Ti surfaces significantly reduced the adhesion of specific periodontal bacteria such as *Fusobacterium nucleatum* and *P. gingivalis*.

Evaluating the development of biofilms generated by co-culturing *P. aeruginosa*, *S. aureus*, and *S. epidermidis* on Ti surfaces, it was found that saliva pellicle allowed greater *P. aeruginosa* strain development compared to *S. aureus* and *S. epidermidis* growth. The finding that *P. aeruginosa* growth is promoted by the salivary pellicle can be attributed to the binding affinity observed between its pili and adhesins and salivary cystatin and mucins^53,54^, which are important components of the salivary pellicle. This is consistent with previous findings showing that preconditioning titanium surfaces with saliva facilitates bacterial adhesion and biofilm development by *P. aeruginosa*^20^.

The precedent that extracellular products of *P. aeruginosa* can inhibit the growth of *S. epidermidis* and *S. aureus* in cocultures^55^ could explain what was observed in the images obtained by SEM in the present investigation, a predominance of adherent bacilli on the experimental and control Ti surfaces. Previous studies have shown that *P. aeruginosa* secretes extracellular products that inhibit the colonization of potential competitors, among them *S. epidermidis*^55^. These extracellular products include 2-heptyl-4-hydroxyquinoline N-oxide, which exhibits significant anti-staphylococcal activity^56^, besides multiple quinolones/quinolines^57^. These quinolones are packaged in extracellular membrane vesicles to directly kill *Staphylococcus* species^58^. This finding was confirmed by DNA-DNA hybridization analysis; the bacterial strain colonizing the experimental substrates was mainly *P. aeruginosa*, followed by *S. aureus*, while the presence of *S. epidermis* could not be detected.

While the literature reports that *S. aureus* and *S. epidermidis* can proliferate abundantly on Ti and its alloys when found together^59^, there are few reports evaluating biofilm formation of these bacteria together with *P. aeruginosa*, individually or in co-culture, on titanium surfaces, and even fewer on titanium surfaces conditioned with salivary pellicle. When the adhesion of the same bacterial strains tested here was evaluated on conventional Ti^60^ with a contact angle of ~70.6, close to the contact angle values of the experimental surfaces in this study: ~81.9°, a fairly close number of cells of each of the tested bacteria, *S. aureus*, *S. epidermidis* and *P. aeruginosa*, was observed, the latter being the strain that showed the lowest affinity for Ti substrates, in the study cited above^60^, the Ti surfaces were not conditioned with the SP.

While the present experimental model establishes a critical baseline for characterizing colonization dynamics on pure titanium substrates, certain methodological constraints must be weighed to properly contextualize the findings. A primary limitation lies in the reliance on a single reference strain of *P. aeruginosa*. Given the profound intraspecific variability that characterizes this pathogen, manifesting in divergent exopolysaccharide production, antibiotic tolerance profiles, and overall virulence expression^61^, extrapolating these observed interaction patterns to the broader species level introduces inherent information uncertainty. Clinical isolates, particularly those adapted to the demanding microenvironments of peri-implant pockets, frequently exhibit adaptive and metabolic mechanisms fundamentally distinct from those of standardized laboratory strains.

Equally important is the structural design of the co-culture assays, which were executed utilizing a fixed initial inoculum ratio. In the highly competitive arena of the oral microbiome, spatial organization, metabolic crosstalk, and ultimate biofilm maturity are acutely sensitive to the initial proportional abundance of the colonizing taxa. Restricting the experimental parameters to a single starting ratio provides a valuable mechanistic snapshot, yet it precludes a comprehensive mapping of how shifting competitive pressures might alter the final community architecture under different conditions. A further limitation of the present study is the reliance on a static *in vitro* biofilm model. Within the dynamic environment of the oral cavity, biofilms are subjected to continuous hydrodynamic shear forces from salivary flow, crevicular fluid turnover, and mechanical disruptions. These mechanical forces significantly dictate initial adhesion strengths and the subsequent maturation of the extracellular matrix. Consequently, interpreting these static adhesion profiles must be done with the understanding that they provide a foundational mechanistic view, rather than a direct translation to the complex biophysical realities of the oral cavity.

To capture the true complexity of polymicrobial implant infections, future research must integrate this wider array of clinical isolates, pathological salivary substrates, and systematically titrated inoculum proportions. Moreover, transitioning from standard culture-dependent quantification to advanced fluorescence-based modalities is essential. Incorporating confocal laser scanning microscopy alongside flow cytometry will allow for a high-resolution, three-dimensional assessment of cellular viability, spatial distribution, and matrix architecture. Pursuing these analytical avenues will substantially refine our understanding of the dynamic interplay between salivary pellicle conditioning, interspecies antagonism, and the physical titanium.

Beyond methodological variables, evaluating bacterial adhesion on biomedical devices must rigorously consider the conditioning pellicle. Rapidly adsorbed from host fluids, this proteinaceous layer fundamentally redefines the physicochemical nature of the biomaterial interface. The historical tendency to attribute adhesion patterns solely to the intrinsic “affinity” of specific strains for bare titanium is an oversimplification; it overlooks how the pellicle dictates colonization by providing new receptor sites and altering surface thermodynamics. Consequently, any evaluation of anti-biofilm strategies that ignores this inevitable salivary layer risks producing clinically irrelevant conclusions. Advancing the field requires abandoning imprecise concepts of direct bacterial affinity in favor of an evidence-based approach that recognizes the biologically conditioned interface as the true determinant of susceptibility to infections.

## Conclusions

The data indicate that the presence of salivary pellicle (SP) on commercially pure titanium (Ti) substrates fundamentally alters the dynamics of biofilm formation. At the individual species level, the presence of SP significantly promotes the development of *P. aeruginosa* biofilms on Ti surfaces, highlighting a possible mechanism by which this pathogen can establish itself on dental implants or other Ti-based medical devices.

In contrast, the SP substantially inhibits biofilm development by *S. epidermidis*. This finding suggests that the pellicle may offer a protective or competitive advantage against certain common colonizers, thereby altering the initial colonization landscape.

Finally, when exposed to a co-culture, the SP decisively and preferentially favors the proliferation of *P. aeruginosa*. This preferential growth occurs at the expense of *S. aureus* and, even more notably, *S. epidermidis*.

In summary, the salivary pellicle should be recognized as an active conditioning layer with a distinct modulatory effect on Ti metal surfaces. While it actively restricts the adhesion, spatial coverage, and viability of staphylococcal strains (*S. aureus* and *S. epidermidis*), it remains an optimal substrate for *P. aeruginosa* colonization. This selective permissiveness, selectively favoring the growth of specific pathogens while restricting other prominent pathogenic colonizers, provides critical insights into the dysbiotic shifts driving implant-associated infections.

## Supplementary Information

Below is the link to the electronic supplementary material.

Supplementary Material 1

Supplementary Material 2

Supplementary Material 3

Supplementary Material 4

## Data Availability

The data that support the findings of this study are available from the corresponding author upon reasonable request.
